# Capacity-building and continuing professional development in healthcare and rehabilitation in low- and middle-income countries—a scoping review protocol

**DOI:** 10.1186/s13643-023-02188-3

**Published:** 2023-02-23

**Authors:** Dalton Deprez, Angela J. Busch, Paola Andrea Ramirez, Eliany Pedrozo Araque, Julia Bidonde

**Affiliations:** 1 School of Rehabilitation Science, University of Saskatchewan, Saskatoon, Canada; 2 Santander, Colombia; 3 Universidad de Santander, Facultad de Ciencias Médicas y de la Salud, Bucaramanga, Santander, Colombia; 4 Norwegian Institute of Public Health, Skøyen, PO Box 222, N-0213 Oslo, Norway

**Keywords:** Human resources for health, Medical education, Resource-limited, Capacity-building, Continuing professional development, Inter-professional training, Health education and promotion, Health systems, Clinical mentorship, Recursos humanos en salud, Educación médica, Desarrollo de capacidades, Desarrollo profesional continuo, Entrenamiento inter-profesional, Promoción y educación de la salud, Sistemas de salud, Tutoría clínica

## Abstract

**Background:**

A recent world health report suggests that there is a growing rehabilitation human resource crisis. This review focuses on the capacity-building needed to meet present and future rehabilitation challenges in low- and middle-income countries (LMICs). Capacity-building is the process by which individuals and organizations obtain, improve, and retain the skills, knowledge, tools, equipment, and other resources needed to do their jobs competently. The objectives of this review are (1) to determine how capacity-building has been defined, implemented, and evaluated in LMICs and (2) to provide an overview of the effectiveness of capacity-building initiatives.

**Methods:**

In the first of seven stages, we will refine and delimit the research. Then, we will identify relevant studies by searching five biomedical databases, two rehabilitation databases, three regional databases, and three databases of gray literature. Two independent reviewers will then select the studies using a priori selection criteria. We will exclude incomplete records, records published prior to 2000 for databases and 2010 for gray literature, and records written in languages other than English or Spanish. We will also exclude records focusing on entry-to-practice programs in academic settings. For Objective 1, using qualitative analysis software, we will extract and analyze text from included records that define or explains capacity building. For Objective 2, using an online file-sharing platform, one reviewer will extract data describing the effectiveness of capacity-building interventions and a second reviewer will verify the accuracy, with disagreements resolved by consensus. The results will be collated using tables and charts. After synthesizing the results, we will discuss the practicality and applicability of the findings with partners from Honduras and Colombia. We will use several formats and venues including presentations and publications in English and Spanish to present our results.

**Discussion:**

To our knowledge, this will be the first attempt to systematically identify knowledge of capacity-building and rehabilitation in LMICs. This scoping review results will offer unique insights concerning the breadth and depth of literature in the area. It is anticipated that results from this scoping review will guide efforts in future capacity-building efforts in rehabilitation in LMICs.

**Review registration:**

Busch AJ, Deprez D, Bidonde J, Ramírez PA, Araque EP. Capacity building and continuing professional development in healthcare and rehabilitation in low- and middle-income countries—a scoping review. 2021. https://doi.org/10.17605/OSF.IO/7VGXU.

**Supplementary Information:**

The online version contains supplementary material available at 10.1186/s13643-023-02188-3.

## Resumen

**Introducción:**

La literatura mundial sugiere que existe una creciente crisis de recursos humanos en el área de rehabilitación. Esta Revisión Sistemática Exploratoria se centra en el desarrollo de capacidades en el área de rehabilitación en países de ingresos bajos y medianos (PIBM). El desarrollo de capacidades es el proceso mediante el cual las personas y las organizaciones obtienen, mejoran y retienen las habilidades, el conocimiento, las herramientas, el equipo y otros recursos necesarios para realizar su trabajo de manera competente.

**Objetivos:**

Determinar cómo se ha definido, implementado y evaluado el desarrollo de capacidades en rehabilitación en los PIBM; y proporcionar una síntesis sobre la eficacia de las iniciativas de desarrollo de capacidades en rehabilitación en los PIBM.

**Métodos:**

En la primera de siete etapas, refinaremos las preguntas de la investigación. Luego, identificaremos estudios relevantes mediante la búsqueda de cinco bases de datos y tres bases de datos de literatura gris. Dos revisores en forma independiente seleccionarán los estudios utilizando criterios definidos a priori. Excluiremos registros (artículos y otra literatura) incompletos, publicados antes de 2000 para bases de datos y 2010 para literatura gris, y escritos en idiomas que no sean inglés o español. También excluiremos registros que sobre programas de ingreso a la práctica profesional (académicos). Para el Objetivo 1, extraeremos y analizaremos el texto que define las estrategias/iniciativas de desarrollo de capacidades en rehabilitación utilizando un software de análisis cualitativo. Para el Objetivo 2, un revisor extraerá datos que describen la efectividad de las intervenciones y un segundo revisor verificará la precisión de los datos utilizando una plataforma electrónica. Los desacuerdos entre revisores se resolverán por consenso. Los resultados se presentarán usando tablas y gráficos. Consultaremos con colegas de PIBM sobre la aplicabilidad de los hallazgos. Para la diseminación de resultados, usaremos presentaciones y publicaciones en inglés y español.

**Discusión:**

Hasta donde sabemos, esta será la primera revisión exploratoria para identificar el desarrollo de capacidades en rehabilitación en los PIBM. Se prevé que los resultados de esta revisión guiarán los esfuerzos futuros de desarrollo de capacidades en la rehabilitación de los PIBM.

## Background

Based on global estimates, one billion people are living with disabilities [1]. The socioeconomic effects associated with disability (e.g., poor health outcomes, lower educational achievement, less economic participation) present serious and growing public health and human rights challenges. As stated in the World Report on Disability, “unmet rehabilitation needs can delay discharge, limit activities, restrict participation, cause deterioration in health, increase dependency on others for assistance, and decrease quality of life” [2].

A recent world health report focuses the world’s attention on human resources as the key factor ingredient to successful health systems functioning, as well as highlighting the growing human resource crisis, particularly in low-income countries [1]. The capacity to provide rehabilitation around the world is limited to non-existent and often fails to adequately address the needs of the population. The global rehabilitation human resource shortage (both in quality and quantity) and uneven distribution of human resources especially in rural and remote areas contribute to inequitable access to rehabilitation and health disparities within and across countries [3]. This disparity is most extreme in low- and middle-income countries where the burden of disability is greatest [2]. Scaling up rehabilitation, however, depends on greater awareness and advocacy, increased investment into rehabilitation workforce and infrastructure, and improved leadership and governance structures [4]. Rehabilitation, however, has been typically neglected in the health workforce agenda [5].

Rehabilitation is a health strategy that aims to enable persons experiencing or likely to experience disabilities to achieve and maintain optimal functioning [6]. Rehabilitation services are offered to people experiencing a range of disabilities, including disabilities tied to mobility, vision, hearing, or cognition [6]. For the purpose of this scoping review, we define rehabilitation service providers as (1) rehabilitation professionals who have specialized training in rehabilitation (specialist doctors, nurses, occupational therapists, physiotherapists, respiratory therapists, psychologists, speech and language therapists, prosthetists, orthotists, social workers) and (2) others who provide direct care rehabilitation service (community-based rehabilitation workers, special educators, other key lay health workers who provide care) [7].

The challenges related to training or supporting rehabilitation providers in resource-limited settings are many, and they go far beyond the mere knowledge transfer of clinical skills. Fostering career development, building health rehabilitation capacity and networks, and retention are key components to advance the goal of creating a strong health rehabilitation workforce and knowledge of best practices for patient care and public health, appropriate to resource-limited settings.

Capacity-building is “the process by which individuals, groups and organizations, institutions and countries develop, enhance and organize their systems, resources, and knowledge; all reflected in their abilities, individually and collectively, to perform functions, solve problems, and achieve objectives [8]. We take note of the critique of Potter and Bough [9] who opine that the construct of capacity-building is an overly broad term, and like these authors, we also accept that the term capacity-building is “merely a starting point for investigation and intervention.” Potter and Bough take a system perspective and described a four-layered capacity pyramid consisting of tools, skills, staff and infrastructure, and structures, systems, and roles. The authors emphasize “systemic capacity building would improve diagnosis of sectoral shortcomings in specific locations, improve project/program design and monitoring, and lead to more effective use of resources.”

*Why is important to do this Scoping Review?*A scoping review aims to “map the literature on a particular topic or research area and provide an opportunity to identify key concepts, gaps in the research, and types and sources of evidence to inform practice, policymaking, and research” [10]. A scoping review is relevant to disciplines with emerging evidence, such as rehabilitation science, “in which the paucity of randomized controlled trials makes it difficult for researchers to undertake systematic reviews” [11]. We believe a scoping review on this topic is warranted since clarity is needed on how capacity-building is defined and implemented in the area of rehabilitation. Utilizing a scoping review will also allow us to summarize how the effectiveness of capacity building has been measured and the outcomes of these assessments. Undertaking this review will also allow us to summarize how the effectiveness of capacity building has been measured and to collate the outcomes of these assessments.

## Methods/design

### Aim

The objectives of this scoping review are to (1) determine how capacity-building has been defined, implemented, and evaluated in LMICs and (2) to provide an overview of the effectiveness of capacity-building initiatives in LMICs.

### Study design

The proposed scoping review will be conducted in accordance with Arksey and O’Malley framework [12] methodology to assess and synthesize the evidence in published and unpublished literature on capacity-building and rehabilitation in LMICs. The present scoping review protocol has been registered with the Open Science Framework (registration: osf.io/7vgxu) and is being reported in accordance with the reporting guidance provided in the Preferred Reporting Items for Systematic Reviews and Meta-Analyses for Protocols (PRISMA-P) statement [13] (see Additional file 1). The proposed review will be reported in accordance with the reporting guidance provided in the Preferred Reporting Items for Systematic Reviews and Meta-analyses extension for Scoping Reviews (PRISMA-ScR) [14]. Any amendments made to this protocol when conducting the study will be outlined and reported in the final manuscript.

### Eligibility criteria

We used the SPICE Framework [15] to delimit the research question (see Table 1). SPICE builds upon the PICO acronym (Population, Intervention, Comparison, and Outcomes). This framework offers a step-by-step approach to formulating questions for finding evidence in existing research.

**Table 1:** SPICE framework

**Setting**	We will consider research conducted in any setting (any health care, rehabilitation, or community setting) in low- to middle-income countries
**Population/participants**	This scoping review will consider all published and unpublished studies relevant to rehabilitation providers who have specialized training in rehabilitation (physiatrists, nurses, occupational therapists, prosthetists, etc.) and others with special training in rehabilitation (technical assistants, special educator, lay workers) who work delivering rehabilitation services. If insufficient literature is available, we will expand the scope to include healthcare personnel (any type of staff who work directly with patients) in LMICs
**Intervention**	The scoping review will consider all records that describe capacity-building (synonymous with capacity development) initiatives or strategies in rehabilitation. Capacity-building is defined as the “process of developing and strengthening the skills, instincts, abilities, processes, and resources that organizations and communities need to survive, adapt, and thrive in a fast-changing world.” [16] If we find insufficient literature on capacity-building for rehabilitation providers, we will expand our scoping review to include capacity-building interventions in healthcare more broadly
**Comparator**	This review will consider records that describe a comparator or no comparator
**Evaluation**	We will document the ways in which the service or action has been measured to establish whether it has had a desired effect. We will extract any outcome data measured using a reliable and valid tool related to (a) skills, attitudes, abilities, knowledge in healthcare, or rehabilitation personnel, (b) processes and resources in organizations or institutions, or (c) performance and health in clients

### Study types

This scoping review will accept experimental and quasi-experimental studies, analytical observational studies, descriptive observational studies, qualitative studies, systematic reviews, and text and opinion papers that meet the inclusion criteria. Studies published in English and Spanish language will be included.

### Search strategy

An experienced information specialist will develop and test the search strategy in consultation with the review authors. Keywords and controlled vocabulary terms will be selected to maximise the sensitivity and specificity of the search. The information specialist will be instrumental in choosing and applying search terms to comply with several databases in the health and social sciences. A sample search strategy for one database is provided in Additional file 2. Upon completion, the results from each database will be documented and the references will be de-duplicated manually. References will then be imported to a review software for screening.

We will search all databases from inception to the date of search. We will include a geographical EPOC filter for low- and middle-income countries. This filter, which is based on the World Bank list of countries (2019), classified as low-income, lower-middle-income, or upper-middle-income economies, has been developed by the EPOC Cochrane group in collaboration with the World Health Organization Library and the Campbell Collaboration. As “rehabilitation” is a narrow area to search, we will review the records left behind when the filter is applied. If we find records of interest, we will remove the filter and run the search strategy without it. We will search in the following databases:

- **Biomedical:** PubMed (MEDLINE), Embase Classic+Embase 1947 to present (OVID), Web of Science Core Collection (Clarivate) 1900 to present, Cochrane Library (Wiley) and Epistemonikos.
- **Rehabilitation:** CINAHL (EBSCO) 1937 to present, PEDro (https://www.pedro.org.au/)
- **Regional:** Bireme-Lilacs, Scielo, LA Referencia.

### Searching other sources

Gray literature is defined here as all research work not published in (commercial) or official mainstream literature for example conference proceedings, government reports, global health agency reports, and dissertations. For this scoping review, we will*:*

1. Search the bibliographies of relevant studies and reviews
2. Select an a priori set of global health/rehabilitation/capacity-building associations and their associations’ webpage(s) will be screened for annual reports or findings that these associations produce based on their own research which will be retrieved
3. Sources: OpenGrey, Gray Literature Report, Lens.

### Study selection

The selection criteria will be open, with the only restrictions relating to publication year (2000 to present for research reports and 2010 to present for gray literature); articles including a rehabilitation component with reference or implications to the workforce; and capacity-building specific and/or relatable to health rehabilitation. Table 2 presents the criteria we will utilize.

**Table 2:** Inclusion criteria

Inclusion criteria	Description
Records topic (e.g., reports, articles, editorials)	Records with the topic of rehabilitation (e.g., physiotherapy, occupational therapy, speech-language therapy, speech-language pathology) and capacity-building or similar terms used in the literature such as education or development. This means where a session (short or long) or program (short or long) was scheduled to impart knowledge in rehabilitation
Records setting	Records limited to capacity-building strategies implemented in a LMICs as defined by the World Bank country classification 2020 [17]
Records’ participants	Participants were rehabilitation providers (i.e., individuals with a degree related to a rehabilitation profession) or other individuals involved in rehabilitation (i.e., community/lay health workers)
Study design	Any study design (e.g., systematic review, scoping review, narrative review, randomized trial, case study, case series, qualitative study, program evaluation)

### Exclusion

- Records with no abstract or full text available after exhausting all possible sources.
- Records written in languages other than English and Spanish.
- Date of publication prior to 2000 for research reports and prior to 2010 for gray literature.
- Records focusing on an entry-to-practice program (diploma or degree) in an academic setting.

Following the search, the identified records will be collated and uploaded in Zotero bibliographic software [18] for deduplication. The final unique record set of potentially eligible studies will be exported to Qatar Computing Research Institute’s Internet-based software, Rayyan [19], through which screening of records will be carried out. Prior to the screening, reviewers will pilot the eligibility criteria on a random sample of 15 titles/abstracts and full text, with further pilot rounds if necessary. Duplicate reports from the same study will either be combined if they report different results or one will be excluded if the results are the same.

We have developed a preliminary set of screening probes (see Additional file 3). Following a pilot of the screening probes, each title and abstract will be screened by at least two independent reviewers (DD, AJB, JB, EPA). An initial calibration will be conducted on 5 to 10 randomly selected records to ensure high interrater agreement. The full text of selected literature will then be retrieved, uploaded into an Internet archive (Google Drive), and reviewed for eligibility, independently by two members of the team (DD, AJB, JB, EAP) using a priori eligibility criteria. Reasons for exclusion will be provided in an appendix in the final scoping review report. Disagreements between reviewers at this stage will be resolved by consensus or by a third member of the research team. The results of the search will be reported in full in the final report and presented in a PRISMA flow diagram [13] (see Additional file 4).

### Data extraction

Data extraction forms will be developed a priori by the first author to capture information on each document included in the review. The forms will be piloted by members of the review team and refined based on feedback from the exercise. Team members (DD, AJB JB, EPA) will carry out data extraction in the following manner: data will be extracted by one reviewer and a second reviewer will verify the data for all records. Disagreements between reviewers will be resolved by consensus. We will use online file sharing (e.g., Drop box, Google Drive) to facilitate collaboration during study selection and data extraction.

For data extraction related to Objective 1, the pdf or text version of the included study will be uploaded into a qualitative analysis tool. We will extract blocks of text from the full-text articles dealing with the definition (concepts, frameworks, or models), description (when, where, what, how), and motives (why) which describe rehabilitation capacity building in LMICs for later coding and qualitative analysis using constant comparative methods.

Table 3 shows data we will extract from included studies/records for objective 2.

**Table 3:** Data extraction criteria objective 2

Data to be extracted	Description
Basic characteristics of the record	Authors, year (study ID), study citation, publication source (peer-reviewed journal, non-peer review journals, agency report, thesis)
	Study design (e.g., systematic review, scoping review, narrative review, randomized trial, case study, case series, qualitative study, program evaluation, editorial, commentary, other)
	Country (classified using a filter for LMICs used by EPOC)
	Language
Characteristics of the target audience/participants of the capacity-building intervention:	Profession: nurse, assisting personnel, physician, rehabilitation provider (physical therapist/physiotherapist, occupational therapists, speech-language pathologists, rehabilitation nurses) and others (functional therapists, fono-audiology technicians, lay health worker, community-based rehabilitation worker, paraprofessional)
	Skill mix: specialist or generalist (rehabilitation skills that apply to service users with a wide range of disabilities)
	Practice setting of target audience/participants (acute care facility, long-term acute care facility, inpatient rehabilitation facility, nursing home and assisted living facility, home healthcare, vehicle (e.g., mobile clinics), and outpatient facility, physician office, non-for-profit organization, other)
	Years of experience in health care, in rehabilitation
Characteristics of the capacity-building intervention	Setting of the intervention/strategy/initiative (location, health system, date of the intervention)
	Historical attributes: prior experience or prior program/intervention, co-existing intervention
	Capacity-building intervention/strategy/initiative type (data-driven adjustments to the classification are anticipated; therefore, forms will be modified as needed) 1. Learning plans or professional development plans 2. Continuing education/professional development workshops 3. Mentorship program, clinical skills tutorial 4. Structured or supervised postgraduate clinical skills training 5. Train the trainer 6. Other
	Number of participants, participant-to-faculty ratio
	Characteristics and country of origin of faculty -
	Description of duration/frequency. Note: Will include details regarding 7. Number of sessions 8. Duration of capacity-building events (hours, days, weeks, months, years) 9. Number of cycles
	Capacity-building method/type: 10. Online, in person, correspondence 11. Networking events (conferences) 12. Certificate or non-certificate program 13. Non-credit or credits given for continuing professional education or medical education, or capacity-building
	Content components 14. Theory related to clinical conditions or clinical interventions 15. Practical clinical skill development 16. Training in social support skills for dealing socially challenged clients
	Funding arrangements, subsidization, or registration fees
	Sponsors (government, non-government, or professional learning objectives
	Outcomes: 17. Participant satisfaction 18. Skills, attitudes, abilities, knowledge, competency in setting and achieving goals in healthcare or rehabilitation 19. Health status impacts (physical, emotional, social) related to performance and health in clients (i.e., attributed to participant learning) 20. Stronger community relationships and networking 21. Increased number of community-based opportunities such as work or skills and training 22. Enhanced ability of participants to share their ideas and actions for change Other (any outcome used to demonstrate the impact of the capacity-building intervention)
	Comparator: any comparator, no comparator

### Data presentation and analysis of results

Objective 1—Qualitative analysis using grounded theory methodology [20] will be conducted using QSR International’s NVivo 12 qualitative software [21]. Qualitative methods (coding, categorizing, conceptual ordering, and theorizing) will be used to develop a synthesized model that incorporates elements of why, when, where, what, and how rehabilitation capacity building in LMIC has been implemented or conceived as described in the included studies and reports [22].

Objective 2—Charts and tables will be used to display the results. Results will be classified based on the key variables: study design, program type, program objectives, program audience, tools used to evaluate outcomes. Data will be aggregated to present an overview of the included studies. We will identify studies for later quality assessment.

### Consulting

This stage of the process, which involves engaging consultants who have expertise in the topical area, is classified as optional by experts [9, 15]. If time allows, the preliminary findings will be discussed with an advisory team made up of partners (i.e., rehabilitation providers, administrators, and academics) from Honduras, Colombia, and Canada. This step will help to evaluate the practicality and applicability of interventions. We will consult with our partners regarding potential avenues for knowledge dissemination.

### Disseminating the knowledge

Although not part of the Arksey and O’Malley framework [15], we believe it is important to make the content of this scoping review available to those involved in rehabilitation education, educational institutions, professional association involved in rehabilitation in low resources, and local, regional, and national governments. The goal of this dissemination is to increase awareness of the literature and to help make evidence-informed choices for capacity-building of the healthcare rehabilitation workforce in low-resource settings. We will seek out global health and rehabilitation forums as a venue to distribute our results. We will distribute a plain language summary report to groups and organizations working in rehabilitation, disability, and global health in low-resource settings. In addition to presentations at research conferences in Honduras and Colombia (Spanish), we plan to write two scientific publications (one in English and one in Spanish). At this point, we have selected a variety of modes and venues including the following:

- Presentation at the Knowledge Translation Poster Day—2022, School of Rehabilitation Science, University of Saskatchewan (English).
- Publish summaries (English and Spanish) on the Red de Rehabilitadores de las Americas Network website [23].
- Develop teaching material from this scoping review (e.g., case study) to be used in the introduction to research course(s) in occupational therapy program(s) involving undergraduate students at Universidad de Santander.
- As part of the RRA-NRWA collaboration with Honduras and Colombia, the results will be shared as part of a researcher-in-residence approach [24] with rehabilitation centers and academic forums like CRILA (rehabilitation center) or the Universidad Autonoma de Honduras functional therapist program representatives.

## Discussion

Since the start of our work in Honduras in 2015, we have identified a paucity of publications pertaining to capacity-building in rehabilitation in LMICs. A scoping review was chosen for its utility in mapping major concepts across a diversity of literature to provide an overview of the degree, scope, and nature of research activities in a broad topic area, as well as to identify gaps in evidence. One of the strengths of this protocol is that it was co-designed with LMICs colleagues (EAP, PAR); and four of the authors have worked in this field in LMICs (AJB, JB, EPA, PAR). It is expected that the findings of this review will provide evidence on the breadth of the rehabilitation capacity-building evidence in LMICs.

The results of this review will also inform further stages of our work in Honduras, including the content and delivery of future capacity-building activities, and can be used to inform new scholarship by other researchers interested in continuing education and rehabilitation in LMICs. Furthermore, through publishing this research protocol, we encourage rehabilitation providers, scholars, researchers, policymakers, and consumers to start the conversation about capacity-building on rehabilitation to strengthen health systems and services and responses to needs.

The review will extend and progress the current knowledge on developing or providing capacity-building initiatives in a meaningful and effective way. The results of the review have the potential to inform local policy discussions. This scoping review is constrained to English and Spanish records which limits the external validity of the findings. Nevertheless, to our knowledge, this is the first scoping review in the area.

### Limitations

Articles will be excluded based on language which could be a source bias. We may not find enough articles to achieve data saturation (Objective 1), which will be a limitation but also a finding on its own.

## Supplementary Information

**Additional file 1. **PRISMA-P Checklist. Reporting of items for a systematic review.

**Additional file 2. **Medline Search Strategy

**Additional file 3. **Screening Probes. Probes for screening records at both an abstract level and full-text level.

**Additional file 4. **PRISMA-P Flow Diagram. Flow diagram template depicting inclusion and exclusion of records throughout the research project.

## Data Availability

The datasets used and/or analyzed during the current study are will be available from the corresponding author on reasonable request.

## Abbreviation

LMICs: Low- and-middle income country(ies)
