# Supplementary material for: Capacity-building and continuing professional development in healthcare and rehabilitation in low- and middle-income countries—a scoping review protocol
Source: Syst Rev. 2023 Feb 23;12:22. doi: 10.1186/s13643-023-02188-3 (PMC9948347; doi:10.1186/s13643-023-02188-3)
Supplement: Supplementary file 2 — Additional file 2. Medline Search Strategy [file 13643_2023_2188_MOESM2_ESM.docx]

**Additional File X – Search Strategy**

|  | **Medline (PUBMED)** |
| --- | --- |
| 1 | "Rehabilitation"[MeSH Terms] OR "Rehabilitation Research"[MeSH Terms] OR "rehabilitat*"[Title/Abstract] |
| 2 | "capacity building"[MeSH Terms] OR "capacity building"[Title/Abstract] OR "building capacity"[Title/Abstract] OR "institutional capacity"[Title/Abstract] OR "capacity development"[Title/Abstract] |
| 3 | ("Rehabilitation"[MeSH Terms] OR "Rehabilitation Research"[MeSH Terms] OR "rehabilitat*"[Title/Abstract]) AND ("capacity building"[MeSH Terms] OR "capacity building"[Title/Abstract] OR "building capacity"[Title/Abstract] OR "institutional capacity"[Title/Abstract] OR "capacity development"[Title/Abstract]) |
| 4 | ("Rehabilitation"[MeSH Terms] OR "Rehabilitation Research"[MeSH Terms] OR "Rehabilitation"[Title/Abstract]) AND "competenc*"[Title/Abstract]) |
| 5 | ((("Rehabilitation"[MeSH Terms] OR "Rehabilitation Research"[MeSH Terms] OR "rehabilitat*"[Title/Abstract])) AND (("capacity building"[MeSH Terms] OR "capacity building"[Title/Abstract] OR "building capacity"[Title/Abstract] OR "institutional capacity"[Title/Abstract] OR "capacity development"[Title/Abstract]))) OR (("Rehabilitation"[MeSH Terms] OR "Rehabilitation Research"[MeSH Terms] OR "Rehabilitation"[Title/Abstract]) AND "competenc*"[Title/Abstract])) |
