# Supplementary material for: Capacity-building and continuing professional development in healthcare and rehabilitation in low- and middle-income countries—a scoping review protocol
Source: Syst Rev. 2023 Feb 23;12:22. doi: 10.1186/s13643-023-02188-3 (PMC9948347; doi:10.1186/s13643-023-02188-3)
Supplement: Supplementary file 3 — Additional file 3. Screening Probes. Probes for screening records at both an abstract level and full-text level. [file 13643_2023_2188_MOESM3_ESM.pdf]

A. *Probes for screening at the title/abstract level*
Criterios de cribado para título y resumen

1.Does the title and/or abstract include the term rehabilitation or term(s) pertaining to a specific rehabilitation discipline?<br><br>¿El título y / o resumen incluye el término rehabilitación o término (s) pertenecientes a una disciplina de rehabilitación específica?	If yes, move to step 2,<br>If no, exclude<br><br>Si es así, continúe con el paso 2;<br>si su respuesta es no, excluya
2.Does the title and/or abstract include term(s) pertaining to capacity building?<br><br>¿El título y / o el resumen incluyen términos relacionados con la creación o desarrollo de capacidades?	If yes, include at the Title/Abstract level,<br>If no, exclude<br><br>En caso afirmativo, incluya,<br>Si su respuesta es no, excluya

B. Probes for screening at the full-text level for both objectives #1 & #2
Criterios de cribado de texto complete para objetivos #1 y#2

3.LMIC or equivalent setting?<br><br>El estudio fue conducido en un país de bajos o medianos ingresos o un ambiente similar	If yes, move to step 4,<br>If no, exclude<br><br>Si su respuesta es "si", siga al paso 4<br>Si su respuesta es "no", excluya
4.Other than an entry-to-practice program?<br><br>El estudio reporta un programa diferente a los programas de inicio de practicas	If yes, move to step 5,<br>If no, exclude<br><br>Si su respuesta es si, siga al paso 5<br>Si su respuesta es no, excluya
5.Focus is rehabilitation workforce or service?<br><br>El foco del estudio son los individuos en el área o servicios de rehabilitación?	If yes, move to step 6<br>If no, exclude<br>Si su respuesta es si, siga al paso 6<br>Si su respuesta es no, excluya

6.Includes a conceptual discussion?<br><br>El estudio incluye una discusión conceptual?	If yes, include for objective #1and continue to step 7,<br>If no, exclude for Objective #1 and continue to step 8<br><br>Si su respuesta es si, incluya para objetive #1 y siga a paso 7<br>Si su respuesta es no, excluya para objetivo #1 y siga a paso 8
7.Capacity building intervention implemented?<br><br>El estudio incluye una intervención/estrategia de desarrollo de capacidades?	If no, exclude for objective #2,<br>If yes, continue to step 8<br><br>Si su respuesta es no, excluya por objetivo #2,<br>Si su respuesta es si, continúe a paso 8
8.Outcome data is reported?<br><br>El estudio tiene datos reportados de las variables de interés	If no, exclude for objective #2,<br>If yes, include for objective #2<br><br>Si su respuesta es no, excluya para objetivo #2,<br>Si su respuesta es si, incluya para objetivo #2
